# Revision of the green lacewing subgenus Ankylopteryx (Sencera) (Neuroptera, Chrysopidae)

**DOI:** 10.3897/zookeys.543.6476

**Published:** 2015-12-09

**Authors:** Laura C.V. Breitkreuz, Shaun L. Winterton, Michael S. Engel

**Affiliations:** 1 Division of Entomology, Natural History Museum, and Department of Ecology & Evolutionary Biology, 1501 Crestline Drive – Suite 140, University of Kansas, Lawrence, Kansas 66045-4415, USA; 2 California State Collection of Arthropods, California Department of Food & Agriculture, 3294 Meadowview Road, Sacramento, California 95832-1448, USA; 3 Division of Invertebrate Zoology, American Museum of Natural History, Central Park West at 79th Street, New York, New York 10024-5192, USA

**Keywords:** Ankylopterygini, Australasia, Oriental, Chrysopinae, lacewing, semiochemicals, taxonomy

## Abstract

The Australasian and Oriental green lacewing subgenus Ankylopteryx (Sencera) Navás (Chrysopinae: Ankylopterygini) is examined and its diversity and placement among other members of the tribe Ankylopterygini is discussed. After study of specimens spanning the full distribution and anatomical range of variation for the subgenus, all prior putative species, resulting in the sole valid species are newly synonymized, Ankylopteryx (Sencera) anomala (Brauer). Accordingly, the following new synonymies are established: *Sencera scioneura* Navás, **syn. n.**, *Sencera feae* Navás, **syn. n.**, and *Sencera exquisita* Nakahara, **syn. n.** [all under the name Ankylopteryx (Sencera) anomala]. A lectotype is newly designated for Ankylopteryx (Sencera) anomala so as to stabilize the application of the name. To support our hypotheses, the wing and general body coloration as well as the male genitalia are reviewed. We elaborate on the possibility of Ankylopteryx (Sencera) anomala being nothing more than an autapomorphic species of *Ankylopteryx* Brauer, as it was originally described. The species is not sufficiently distinct to warrant recognition as a separate subgenus within the group, and most certainly not as its own genus as has been advocated by past authors. Nonetheless, we do not for now go so far as to synonymize the subgenus until a more extensive phylogenetic analysis is undertaken with multiple representative species from across *Ankylopteryx* and other ankylopterygine genera. Lastly, we comment on the biology of Ankylopteryx (Sencera) anomala in terms of the attraction of males to methyl eugenol and on the widespread practice of splitting within Chrysopidae.

## Introduction

**Figure 1.**
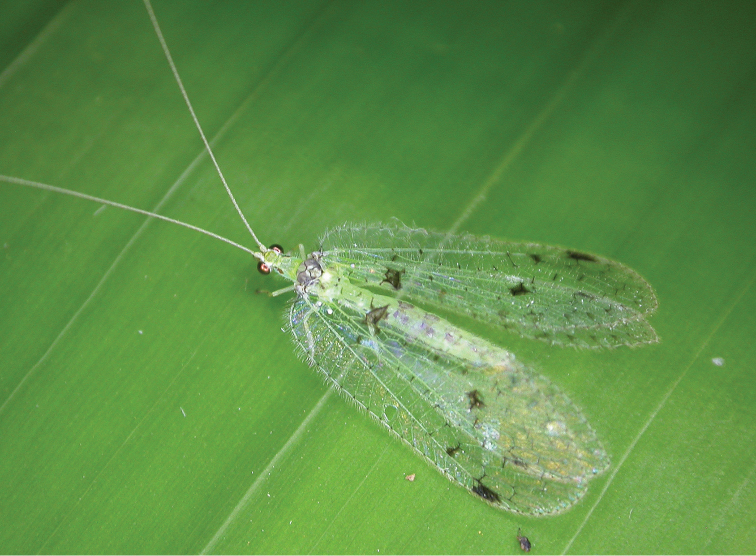
Photograph of live male of Ankylopteryx (Sencera) anomala (Brauer) from Chiang Mai, Thailand (photograph by S.L. Winterton).

The green lacewings (Chrysopidae) of the Australasian and Oriental regions comprise a diverse, yet poorly studied fauna (e.g., Nakahara 1955; New 1980; Brooks 1983, 1997; Tsukaguchi 1995; Yang et al. 2005; Winterton et al. 2012), with several genera in need of revision. One such genus is *Ankylopteryx* Brauer, including the subgenus *Sencera* Navás (Fig. 1) (Brooks and Barnard 1990; Brooks 1997). This subgenus is rarely encountered but distributed throughout the Orient and Australasia, ranging from Nepal to Vanuatu. *Sencera* was proposed by Navás (1924) to separate the species *Sencera scioneura* Navás from the remainder of the genus *Ankylopteryx* [this species is herein considered a synonym of *Ankylopteryx anomala* (Brauer)]. The description of the genus was based on the lack of the intramedial (*im*) cell in the forewing despite the fact that, apart from this isolated venational difference, all other characters were identical with *Ankylopteryx*. Two further taxa were added later — *Ankylopteryx feae* (Navás) in 1929 and *Ankylopteryx exquisita* (Nakahara) in 1955 (both originally proposed within *Sencera*) (Navás 1929; Nakahara 1955), bringing the diversity to four nominal species and almost exclusively known only from their type series. The late Nathan Banks (1868–1953) mentioned, but never published, his speculation of the possible synonymy of *Ankylopteryx anomala* and *Ankylopteryx scioneura* (Banks’ suspicion was specifically mentioned by Nakahara 1955), but otherwise there has been no evaluation of the circumscription of species within *Sencera* beyond Brooks and Barnard (1990), Tsukaguchi (1995), and Yang et al. (2005).

Here we present a brief review of the subgenus based on the most extensive sampling of these rare lacewings, and elaborate and expand upon Nathan Banks’ suspicions. Indeed, others have also noted similarities which, when taken into a broader context, suggest that it is not only the species that are suspect but the subgenus as a whole. Brooks (1983) recognized the striking similarity between *Ankylopteryx* *s.str.* and *Sencera*, emphasizing genitalic characters that united the two. He elaborated on their relationship and later demoted *Sencera* to subgeneric rank within *Ankylopteryx* (Brooks and Barnard 1990). The fact that some Oriental and African *Ankylopteryx* have a dramatically reduced *im* cell (Brooks 1983; Brooks and Barnard 1990; Tsukaguchi 1995) suggests the possibility that such species might form a grade relative to the loss observed in *Sencera*. Indeed, Tsukaguchi (1995) considered *Sencera* as a junior synonym of *Ankylopteryx*, and this synonymy should likely be re-established. Accordingly, it is important to re-evaluate diversity within *Sencera* and to determine whether further putative apomorphies for the group might be discovered. In addition to the few specimens of the four species that are known, we have examined individuals from various geographic localities. We amassed the largest sampling of this rare group, newly documenting the observed variation and providing a revised description and circumscription of the included species. This is done in the hopes that it will enhance our understanding of the patterns of variation across their range as well as ultimately permit revised hypotheses of relationship (Grimaldi and Engel 2007).

## Material and methods

The higher classification followed is that of Brooks and Barnard (1990), and the morphological terminology used is that of New (1989), Brooks and Barnard (1990), and Aspöck and Aspöck (2008), the latter for genitalic structures. Measurements were made using an ocular micrometer. Photomicrographs were prepared using a Canon EOS 7D digital camera attached to an Infinity K-2 long-distance microscope lens, and then arranged in Adobe Photoshop CS5. Dissections of the genitalia were made under an Olympus microscope. The terminalia were cleared in 10% KOH, washed twice in dH_2_O, and stained with chlorazol black in 80% ethanol. Line drawings were prepared in Adobe Illustrator CS5.

In total we examined 49 specimens during the course of this study, which are deposited in the following institutions and were provided through the generosity of the named curators:

NHML The Natural History Museum, London, United Kingdom (Ben Price)

CAS California Academy of Sciences, San Francisco, California, USA (Norman Penny)

CSCA California State Collection of Arthropods, California Department of Food & Agriculture, Sacramento, California, USA

High-resolution photographs of historical type material that was otherwise not available for loan were contributed by the following:

NHMW Naturhistorisches Museum, 2. Zoologische Abteilung, Vienna, Austria (Harald Bruckner), for *Ankylopteryx anomala*.

NMNS National Museum of Nature and Science, Department of Zoology, Tsukuba-shi, Ibaraki, Japan (Utsugi Jinbo), for *Ankylopteryx exquisita*.

MCSN Museo Civico di Storia Naturale “Giacomo Doria”, Genoa, Italy (Maria Tavano), for *Ankylopteryx feae*.

ZMB Museum für Naturkunde, Berlin, Germany (Lukas Kirschey, Michael Ohl), for *Ankylopteryx scioneura*.

## Systematics

### Tribe Ankylopterygini Navás  Genus *Ankylopteryx* Brauer

#### Subgenus Sencera

Taxon classificationAnimaliaNeuropteraChrysopidae

Navás

Sencera Navás 1925: 26. Type species: *Sencera scioneura*Navás 1925, by original designation. Brooks 1983: 6 [keyed as genus]; Brooks and Barnard 1990: 157 [demoted to subgeneric rank, redescribed]; Tsukaguchi 1995: 10, 122 [synonymy with *Ankylopteryx*]; New 2003: 92 [keyed as subgenus of *Ankylopteryx*]; Yang et al. 2005: 51 [keyed as subgenus of *Ankylopteryx*].

##### Diagnosis.

**Figure 2.**
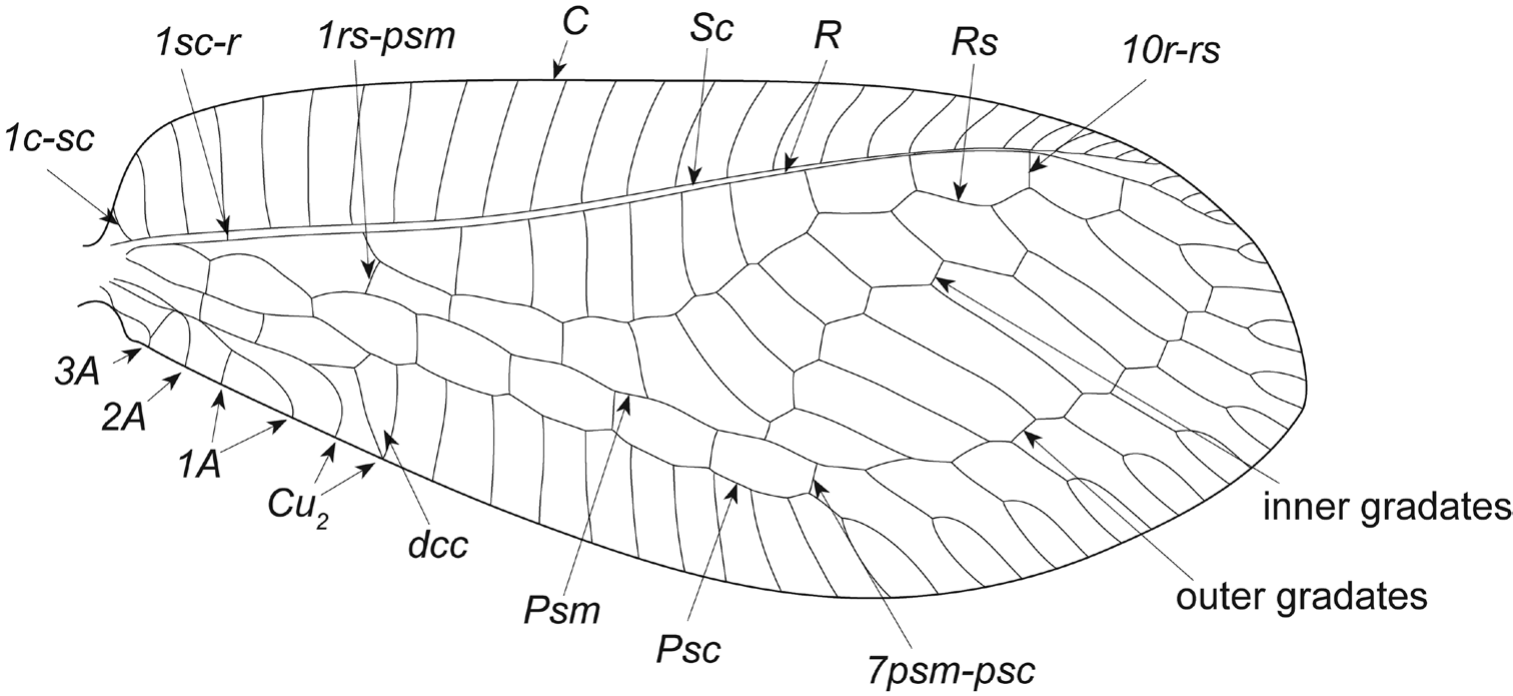
Line drawing of forewing of Ankylopteryx (Sencera) anomala (Brauer).

The subgenus *Sencera* differs from *Ankylopteryx* *s.str.* only in the absence of the forewing ‘*im*’ cell (Fig. 2). Brooks and Barnard (1990) mentioned further differences in the forewing length, ratio of forewing length and width, and ratio of head and compound eye widths but these are all overlapping with those values for species of *Ankylopteryx* *s.str.* The same is true for the slight color differences noted between the two.

#### Ankylopteryx (Sencera) anomala

Taxon classificationAnimaliaNeuropteraChrysopidae

Brauer

Figs 1 , 2 , 3–6 , 7–10 , 11–13 , 14

Ankylopteryx anomala Brauer 1864: 901. Lectotype ♂, NHMW (*visum*).
Sencera scioneura Navás 1924 [1925]: 27. Holotype ♂, ZMB (*visum*). **Syn. n.**
Sencera feae Navás 1929: 371. Holotype ♂, MCSN (*visum*). **Syn. n.**
Sencera feai Navás 1930: 23 [*lapsus calami pro Sencera feae*Navás 1929].
Sencera exquisita Nakahara 1955: 143. Holotype ♂, NMNS (*visum*). **Syn. n.**
Ankylopteryx (Sencera) anomala Brauer: Brooks and Barnard 1990: 157 [combination implied].
Ankylopteryx (Sencera) scioneura (Navás): Brooks and Barnard 1990: 157 [combination implied].
Ankylopteryx (Sencera) feae (Navás): Brooks and Barnard 1990: 157 [combination implied].
Ankylopteryx (Sencera) exquisita (Nakahara): Brooks and Barnard 1990: 157 [combination implied]; Yang et al. 2005: 56.
Ankylopteryx exquisita (Nakahara): Tsukaguchi 1995: 131.

##### Diagnosis.

As for the subgenus (*vide supra*).

##### Description.

**Figures 3–6.**
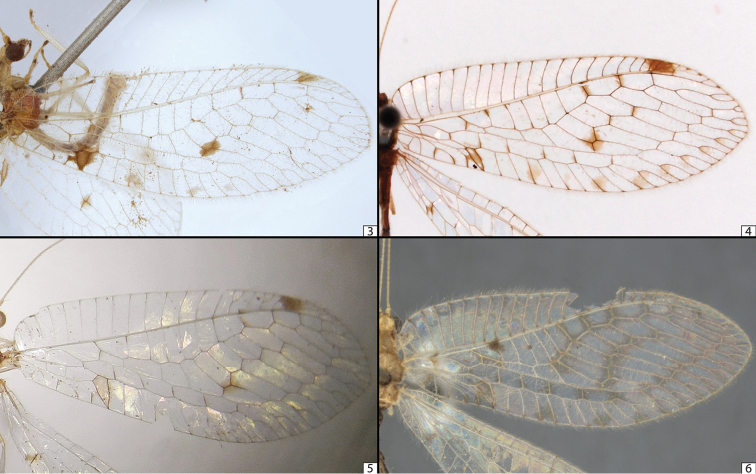
Photographs of forewings of Ankylopteryx (Sencera) anomala (Brauer) **3** Lectotype male from Pulo Milu, Nicobar Islands, India (NHMW) (photograph by Harald Bruckner) **4** Holotype male (mirrored) of ‘*Sencera exquisita* Nakahara’ from Taiwan, China (NMNS) (photograph by Utsugi Jinbo) **5** Holotype male (mirrored) of ‘*Sencera feae* Navás’ from Bhamò, Myanmar (MCSN) (photograph by Maria Tavano) **6** Holotype male (mirrored) of ‘*Sencera scioneura* Navás’ from New Britain, Papua New Guinea (ZMB) (photograph by Lukas Kirschey). All photographs used with permission.

♂. Overall color in live specimens light green with mostly greyish brown and some whitish markings (Fig. 3); in dried specimens green areas appears pale yellow or light brown.

*Head*: vertex smooth, raised and flat; laterally pale green, medially light green, with brown marking medially above toruli [varying in size and intensity of coloration from faint to dark, heart-shaped marking]. Frons smooth and flat; light green to whitish [in some specimens slightly darker than vertex] with small brown marking medially below toruli [varying in size and intensity of coloration from not visible to clearly visible marking (size about distance between toruli)]. Malar space broad; with brown marking extending from mandibular base to lower compound eye margin and epistomal sulcus. Clypeus smooth, slightly raised, indented medially at apical margin; medially light green to whitish, laterally and apically with brown marking. Labrum smooth, flat, apical margin simple without indentation, with brown markings basolaterally and apically. Mandible smooth, apex pointed; dark brown. Maxillary palp light green, fifth palpomere brown. Labial palp light green, third palpomere brown. Gena ventrally flat; light green [in few specimens with brown marking medially]. Scape short and broad (ca. 1.25 times as long as wide); light green to whitish with brown marking laterally [varying in size and intensity of coloration from absent to dark brown longitudinal band]. Pedicel short (ca. 1.1 times as long as wide); light green to whitish. Flagellomeres ca. 2.4 times as long as wide; light green to whitish; setae in 4 rows, long (varying within single flagellomere from as long as flagellum width to twice as long), brown.

*Thorax*: pronotum ca. 0.9 times as long as wide; light green with brown longitudinal marking anterolaterally [varying in size from spot anteriorly to stretching over 2/3 of pronotum and intensity of coloration from almost not visible to dark brown]; setae whitish, long. Meso- and metathorax light green laterally and ventrally, dorsally mostly brown-greyish with some light green and pale green [intensity of brown greyish markings varying]; setae whitish, microsetae dense, long setae sparse. Prescutum with more light green than brown-greyish in some specimens; setae whitish, microsetae dense, long setae sparse. Metascutum with whitish marking; setae whitish, microsetae dense, long setae sparse medioanteriorly. Postmetascutellum light green with small brown-greyish marking anteriorly; setae whitish, microsetae dense, long setae sparse.

*Legs*: light green, fifth tarsomere and pretarsal claws dark brown; most specimens with brown marking mediodistally on pro- and mesotibia [varying in size and intensity of coloration of marking from absent on both legs to well-defined dark brown spots on both legs, marking on mesotibia mostly smaller than on protibia]; setae long, mostly whitish, some brown. Pretarsal claws dilated basally.

**Figures 7–10.**
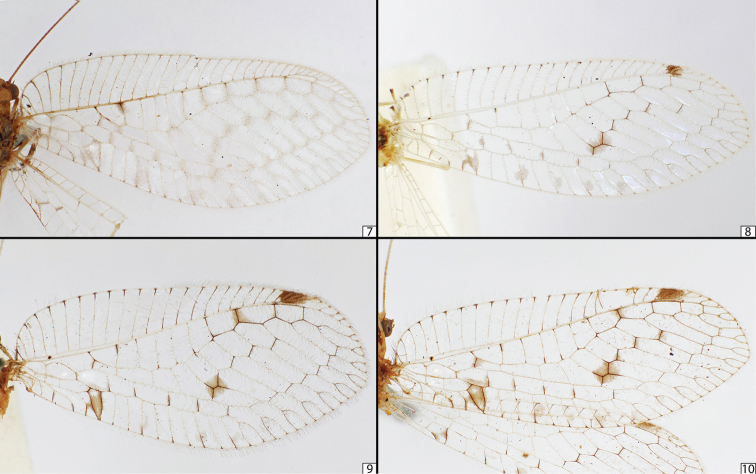
Photographs of forewings of Ankylopteryx (Sencera) anomala (Brauer) showing gradations in wing colouration, from almost unmarked (**7**), to slightly marked (**8**), to more strongly marked (**9**), and ultimately to very strongly marked (**10**). Specimens from Vanuatu (**7**), Brunei (**8**), Myanmar (**9**), and Hainan, China (**10**). All photographs by L.C.V.B.

*Forewing* (Figs 2–10): mean length 10.7 mm; wing ca. 2.6 times as long as wide, slightly pointed apically. Veins mostly pale green [varying from almost all pale green to several veins dark at joints to various veins completely dark]; setae whitish [some setae partially brown, corresponding with wing markings]. Markings on membrane vary from almost absent (with small, faint, light-brown markings) to several dark-brown markings; costal area broad (ca. 0.3 times as wide as total wing width, varying between 0.27–0.33 mm); pterostigma varying from faint light brown (almost absent) to dark brown, extending over 4 crossveins (*2sc-r* – *5sc-r*); *1c-sc* (basal costal crossvein) brown at wing margin; *1sc-r* (bsx) brown; *1rs-m* brown in some specimens; *1r-rs* brown in some specimens. Membrane sometimes with brown marking surrounding *r-rs* (radial crossveins) crossveins (normally *7r-rs* – *10r-rs*), with brown marking in some specimens at *Rs* on a few *r-rs*. Venation as in most Ankylopterygini except *im* cell absent; *Psm* continuous with outer gradates; number of inner gradates varying from 5–7 (number varies also in a single individual, with left and right wings bearing different numbers); veins mostly brown, with brown marking on surrounding membrane of some veins in several specimens; basal inner gradate meeting *Psm*; most specimens with brown marking surrounding base of inner gradates; number of outer gradates varying from 6–8 (varying also between wings in same individual); faint brown coloration on surrounding membrane of outer gradates in a few specimens; *Cu2* and *1A* with brown marking at wing margin in some specimens; *dcc* closed, marked brown [varying in coloration from light to dark brown and in size of marking from not filling the entire cell to extending into the surrounding cells]; 5 *psm-psc* (crossveins between *Psm* and *Psc*) apical of *dcc*, some crossveins with light brown markings surrounding vein in several specimens; light brown markings surrounding some terminal branches of *Psc*; junction of wing margin and veins brown in most specimens, especially in apical half of wing.

*Hind wing*: narrow (ca. 3.5 times as long as wide), apically more strongly pointed than forewing. Veins mostly pale green [varying from almost all pale green to some veins brown at joints, only few veins completely brown]; setae pale [some setae partially brown, corresponding with wing markings]. Costal area narrow (ca. 0.11 times as wide as total wing width). Several *r-rs* (*Rx*) with brown markings surrounding vein (normally *6r-rs* – *9r-rs*) in most specimens, or only brown veins without surrounding marking; number of inner gradates varying from 3–5 (varies also between wings in same specimen); veins mostly brown; basal inner gradate meeting *Psm*; most specimens with brown marking surrounding base of inner gradates; number of outer gradates varying from 4–7 (also varies at times between wings in same specimen); veins mostly brown. Area between *Cu_2_* and basal-most terminal branch *Psc* with brown marking [varying in size and intensity of coloration]. Light brown markings surrounding some terminal *Psm* branches in some specimens; some *psm-psc* with small light brown markings surrounding vein in some specimens, two apicalmost *psm-psc* brown in most specimens; junction of wing margin and veins brown in most specimens, especially in apical half of wing.

*Abdomen*: Terga light green with brown-greyish markings dorsally on terga IV–IX, markings broader on anterior terga. Sterna light green; sterna VIII+IX fused. Setae whitish, microsetae dense, long setae more sparse.

**Figures 11–13.**
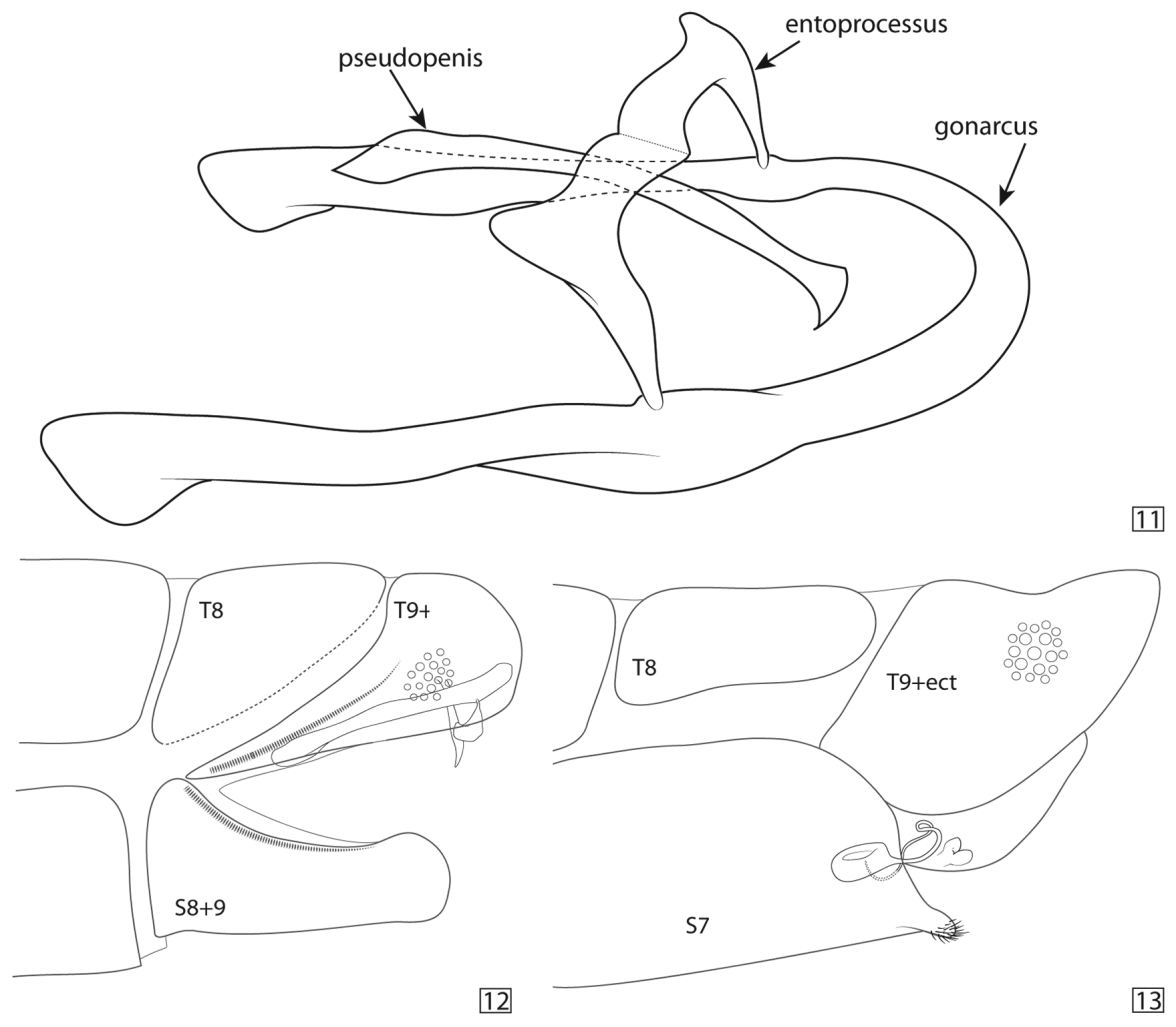
Male and female terminalia of Ankylopteryx (Sencera) anomala (Brauer). **11** Line drawing of male genitalic sclerites **12** Lateral view of male abdominal apex with genitalia **13** Lateral view of female abdominal apex with genitalia.

*Genitalia* (Figs 11, 12): Only gonarcus, entoprocessus, and pseudopenis present. Gonarcus broadened at several locations, especially at apex of lateral arms [variation in general width of gonarcus between specimens; medial arch of gonarcus varying from smooth and only slightly broadened to having broader area forming small horn-like structure]. Entoprocessus loosely attached at narrow connection point to gonarcus; broadened medially, arms meeting medially, forming arch from gonarcus over pseudopenis. Pseudopenis long, subapically broadened and pointed apically [pseudopenis does not stain well in some specimens]. Gonosaccus with few gonosetae.

♀. Characters as in male except terminalia: *Terminalia* (Fig. 13) Sterna VII straight, apically slightly pointed ventrally, with setae at apex. Subgenitale and spermatheca with spermaduct present; subgenitale bilobed apically; spermatheca round (as wide as long); spermaduct coiled, ca. 2 times as long as spermatheca.

##### Measurements.

Based on average from 5 specimens: Head 0.74 times as long as wide; upper distance between compound eyes 1.22 times lower distance between compound eyes; clypeus 0.51 times as long as wide; labrum 0.52 times as long as wide; malar space 1.09 times as long as mandibular base is broad; scape 1.09 times as long as wide; pedicel 1.19 times as long as wide; flagellomeres 3.7 times as long as wide (measured medially on flagellum); thorax 1.43 times as long as wide (measured in dorsal view); pronotum 0.95 times as long as wide (measured in dorsal view); forewing 2.62 times as long as wide; forewing costal width 0.3 times width of forewing; hindwing 3.57 times as long as wide; terga 1.51 times as long as wide (average of third tergum, difficult to measure when dry).

**Lectotype (here designated).** ♂, [India], M, Novara [Reise], 1857–59, Milu, Nicob. (Fig. 3) [this is the first specimen referred to by Brauer (1864) in “Ins. Nicobaricae Milu et Sambelong”, where Milu refers to today’s Pulomilo or sometimes as Pulo Milu, a small island off the north coast of Little Nicobar], deposited in NHMW. We have selected this syntype specimen to serve as the lectotype given the fact that it preserves the most characters (the paralectotype is today in exceedingly poor condition), and better ensures the correct application of the epithet. It should be noted that at some point holotype and paratype labels were placed on Brauer’s series, likely by a curator of the collection as these are newer labels, but these have no standing as Brauer himself never selected an individual to act as the name-bearing type and no subsequent designations of lectotype have ever been published. Thus, those labels have no nomenclatural standing and this is fortunate as the ‘paratypus’ label was placed on the most complete specimen (here selected as the lectotype), and the ‘holotypus’ label on the least well preserved specimen (thus serving the least value to taxonomic stability had they any validity).

##### Paralectotype.

♂, [India], O, Novara Reise, 1857–59, Sambelong, Nicob. [this is the second specimen referred to by Brauer (1864), “Ins. Nicobaricae Milu et Sambelong”, with Sambelong today being the island of Grand Nicobar], deposited in NHMW.

##### Additional material examined.

In addition to the syntype series, a total of 47 specimens available for study (21 ♂♂, 11 ♀♀, 15 sex undetermined), institutional repository and original identification of material indicated in square brackets: **AUSTRALIA**: 1♂, label imprecise: “Australia?”, date unknown; collector unknown [BMNH: originally as *Sencera scioneura*]. **BRUNEI**: 3♂♂, 1 sex indet., June 16^th^ 1984, collector A. Saman, Triencide trap [BMNH: originally as *Sencera anomala*]. **CHINA**: 1 sex indet., Hainan, You Boi, 1911, collector unknown [BMNH: originally as *Sencera exquisita*]. **INDIA**: 1 sex indet., Pirmed, 3400 ft., May 4^th^–6^th^ 1937, collector Travencore [BMNH: indet. #1]. **INDONESIA**: 1♀, Sulawesi, Utaria, October 1985, collector unknown, Project Wallace of the R. Ent. Soc. Lond. [BMNH: originally as *Sencera anomala*]. **MALAYSIA**: 1♂, 2 sex indet., Bettotan near Sandakan, individual dates of July 26^th^, July 30^th^, and August 3^rd^ 1927, collectors C.B.K & H.M.P [BMNH: originally as *Sencera anomala*]; 2♂♂, 1 sex indet., Cameron Highlands, May 22^nd^ 1983, Methyl Eugenol lure trap, collector R.A.I. Drew [CSCA: indet]; 1♂, 3 sex indet., Kedah, nr. Jitra, individual dates of April 4^th^, 10^th^, and 11^th^ 1928, collector H.M. Pendlebury [BMNH: indet. #2]; 6♂♂, Selangor, Gomback, Ulu Gomback Research Station, March 16^th^–17^th^ 2006, 03°19'29"N 101°45'11"E, Steiner trap, Methyl Eugenol, collector T. Dikow [5♂♂ CSCA: indet; 1♂ CAS: indet.]; 1♀, Selangor, Ulu Langat, November 2^nd^ 1981, collector K.R. Tuck [BMNH: indet. #2]. **MYANMAR**: 2♂♂, 1♀, Tenasserim, 1938, collector McLachlan [BMNH: indet. #1]; 1♂, Bhamò, Birmania, vii.1886 [MCSN: holotype of *Sencera feae*]. **NEPAL**: 1♂, 1 sex indet., Chitwan, Sauraha, December 26^th^ 1981 – January 9^th^ 1982, collector L. Jessop [BMNH: originally as *Sencera feae*]. **PAPUA NEW GUINEA**: 1♀, Finschhafen, April 9^th^ 1944, collector E.S. Ross [CAS: indet]; 1♂, Neu-Britannien, Ralum, F. Dahl S., zum Licht sufl., auz Juli 96., 13.12.96 [ZMB: holotype of *Sencera scioneura*]. **SRI LANKA** [Ceylon]: 1♀, Galle, February 10^th^ 1907, collectors Bainbrigge & Fletcher [BMNH: originally as *Sencera feae*]; 1 sex indet., Kottawa, April 24^th^ 1892, collector unknown [BMNH: indet. #1]; 1♀, Nawalapitiya, 1938, collector McLachlan [BMNH: indet. #1]; 1♀, detailed locality unknown, 1938, collector McLachlan [BMNH: indet. #1]. **VANUATU** [New Hebrides]: 1♀, Erromanga, July 1930, collector L.E. Cheesman [BMNH: originally as *Sencera scioneura*]; 2♀♀, Malekula, Ounua, March-April 1929 and May 1930, collector L.E. Cheesman [BMNH: originally as *Sencera scioneura*]. **TAIWAN** [Central Formosa]: 2 sex indet., Suishako, 1911, collector unknown [BMNH: originally as *Sencera exquisita*]; 1♂, Hori Formosa, 5.v.1939, Tomio Kaneko [NMNS: holotype of *Sencera exquisita*]. **THAILAND**: 1♀, Chiang Mai Province, Samoeng Tai, 600m, July 14^th^ 2013, 18.8598°N 98.6507°E, collector S. Winterton [CSCA: indet]; 1♂, 3 sex indet., Trang Province, Khao Chong, October 20^th^–27^th^ and December 1^st^–8^th^ 2008, 7 32’ N 99 47’ E, collectors P. Kongnoo & T. Tongrod [CSCA: indet].

##### Comment.

Although previous authors have alluded to other, putative species in *Sencera* (e.g., New 2003), we cannot confirm any such diversity and all of those forms result from splitting species based on minor variations in wing pattern and coloration. We have found that such patterns are merely variants of a single widespread species. The patterns only superficially appear to be geographically distinct when looking at very small sample sizes from isolated geographic localities (*vide infra*).

## Discussion

### Systematics

We compared the four nominal species in *Sencera* (*Ankylopteryx anomala*, *Ankylopteryx exquisita*, *Ankylopteryx feae*, and *Ankylopteryx scioneura*) as well as several undetermined specimens, some of which were considered putatively new species. Even at first glance over previously determined material it was evident that there is and has been a great amount of confusion when it comes to identifying specimens to one of the original four species. The species in *Sencera* were established on differences in wing coloration (Figs 3–6), some of which overlap, and the original descriptions are not unambiguous, often failing to mention any clear distinction from other species. The wing coloration can vary from multiple large dark areas in the fore and hind wings (Fig. 10) to just barely-visible, pale spots (Fig. 7). As the species are all comparatively rare, suitable samples have been difficult for authors to obtain. With a comparatively larger sample size of 49 specimens (large for a group of four, rarely-encountered taxa!) that span the geographic gaps between the previously isolated localities of the extremes in variation, we discovered that these color patterns cannot be sorted into definite groups. The specimens collected in the easternmost regions (mostly *‘Ankylopteryx scioneura’* from Vanuatu) are generally less colored than specimens in the west and north (*‘Ankylopteryx feae’* and previously undetermined specimens from northern India, or *‘Ankylopteryx exquisita’* from near Hong Kong and Taiwan). In the intervening regions, such as Thailand, specimens show somewhat intermediate wing colorations (e.g., *Ankylopteryx anomala*), and often certain color patterns are unique to a single individual. The same applies for slightly varying sizes in body length and wing width. Ultimately, rather than distinct species there is a continuous variation of wing coloration and width as well as darkness of the body, with numerous overlapping combinations which can to some degree be associated by locality. It is understandable that past researchers who had only seen the extremes of these color forms described them as individual species, and failed to detect the actual continuous variation. The wing coloration does not only vary greatly between but also slightly within specimens — one side having a different degree of coloration than the other. Since wing venation is known to vary between the two sides (Barnard 1984; pers. obs.), it is unclear how reliable small differences in forewing coloration may be for the circumscription of distinct species. Even if, for example, specimens with almost no markings on the forewing would be grouped together, then there remain intermediates that show only scant markings. This is more complicated on the other end of the spectrum, when one individual has strongly marked wings but may lack merely one spot relative to another, virtually identical specimen.

Just as with wing coloration, the intensity of markings on the pronotum and whether a spot is present and the degree of its development on the protibia vary similarly. Some specimens from Brunei lack this protibial spot, in others it is small but present — suggesting that this character is almost as variable as the wing coloration. All other characters are similar to the remaining specimens and this missing spot is not mentioned in any description and does not seem to be indicative of an endemic ‘spotted-protibia species’. More importantly, we dissected the male genitalia from specimens representing the full spectrum of coloration and found no significant differences among them. The genitalia of some specimens are slightly thicker but these are exceedingly small variations and seem to be correlated with body size and degree of pigmentation rather than any boundary between taxa. Accordingly, there are no discrete units identifiable across the variation observed, and our larger sample sizes are indicative of a single, widespread, and variably-colored species. This has served as the basis for our aforementioned synonymies.

Not only has the similarity of those previously recognized species within *Sencera* been striking, but also the dramatic sameness of the subgenus and *Ankylopteryx* *s.str.* As mentioned in the original description of *Sencera*, the only difference between these groups is the absence of the *im* cell (Navás 1924). Given that one of the original species of *Sencera* was originally placed within *Ankylopteryx*, the agreement between the two is obvious, and one can rightly question whether it is worth retaining the former as a distinct group. Both *Ankylopteryx* *s.str.* and *Sencera* possess only a gonarcus, entoprocessus, and pseudopenis (Fig. 11) (Brooks 1983; Brooks and Barnard 1990), and this condition is likely a synapomorphy supporting *Ankylopteryx* *s.l.* Brooks (1983) emphasized the genitalic structure as reflecting the close relationship between *Sencera* and *Ankylopteryx*, and further noted the considerable similarity between them and *Parankylopteryx* Tjeder. The arcessus of *Parankylopteryx* is fused with the gonarcus and not detached (as the ‘pseudopenis’) as in the other two genera. The same author also mentioned several species of *Ankylopteryx* occurring in Africa and the Oriental region that have a small, somewhat reduced *im* cell (e.g., *Ankylopteryx doleschali* Brauer, *Ankylopteryx obliqua* Banks, and *Ankylopteryx decorsei* Navás), and Tsukaguchi (1995) also noted the considerable similarity with some Asian species (e.g., *Ankylopteryx gracilis* Nakahara). These are eminent arguments for the notion that *Sencera* is nothing more than an autapomorphic species of *Ankylopteryx* in which the reduction of the *im* cell has reached its apogee (i.e., complete absence) relative to others in the genus. This position is presently only speculative and so we have avoided formalizing a reinstatement of the synonymy for the generic groups until after it can be tested in a comprehensive cladistic analysis. Nonetheless, Tsukaguchi’s (1995) original synonymy seems to have been prescient.

The synonymy of the four species of *Sencera* discussed here begs mention of an issue common to the taxonomy of lacewings. Within Chrysopidae there are a vast number of species and genera that are characterized by exceptionally small differences in trifling traits by comparison to their closest relatives, and such supraspecific groups are frequently monotypic (Brooks and Barnard 1990; Winterton and Brooks 2002). The trend of often unjustified splitting — describing new species and even genera based on such minimal variations — is not beneficial and complicates research on this interesting family, particularly when many are not correlated with significant differences in the genitalia. The situation is exacerbated when these differences are based on single traits and supporting data are not provided. The history of chrysopid taxonomy is one of consistently reorganizing units into smaller groups, or describing newly discovered variants as new genera without demonstrating the concomitant, reciprocal monophyly of the most similar genus when such a newly described taxon is established. Many taxa should be revisited to address this ongoing issue and ultimately provide a more rigorous classification for evolutionary studies within Chrysopidae (Winterton and Brooks 2002). As it stands, the current taxonomic situation tends to obscure relationships owing to the retention of groups strongly suspected as paraphyletic, and thereby limiting the predictive value of the classification and our understanding of evolutionary phenomena.

### Biology

**Figures 14.**
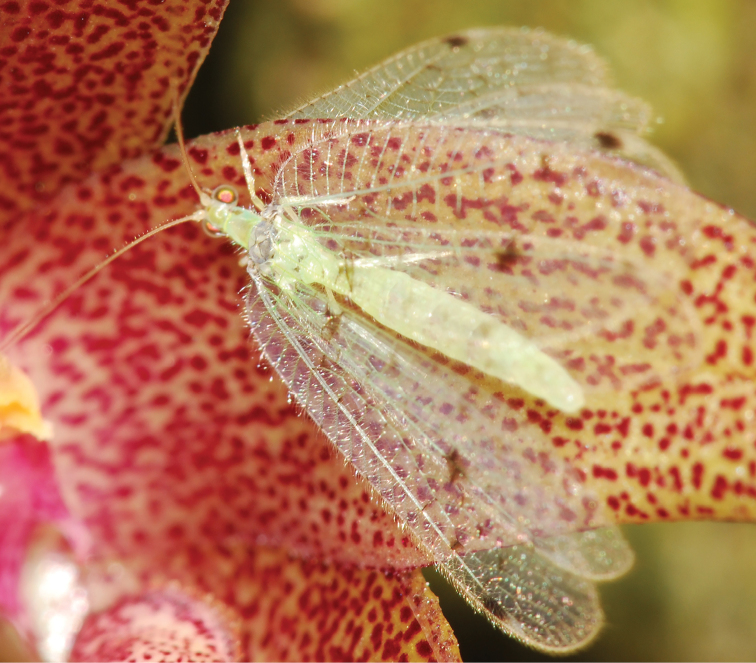
Photograph of a live male of Ankylopteryx (Sencera) anomala (Brauer) from Kuala Lumpur, Malaysia at a flower of an unidentified orchid species of the genus *Bulbophyllum* Thouars (Orchidaceae: Epidendroideae) (photograph by P.T. Ong, used with permission).

Males of *Sencera* are attracted to methyl eugenol (IUPAC: 1,2-Dimethoxy-4-prop-2-en-1-ylbenzene) (Tsukaguchi 1995; Pai et al. 2004; pers. obs.), a phenylpropanoid found in many plants (Tan and Nishida 2012). This suggests a potential for collecting with special baits as has been done with males of the fruit fly genus *Bactrocera* Macquart (Leblanc et al. 2009). Insects attracted to methyl eugenol have been observed to acquire the chemical from different orchid flowers. Along with dacine fruit flies, males of *Sencera* were found on an orchid of the genus *Bulbophyllum* Thouars (Epidendroideae: Podochileae) in high abundance (Fig. 14).

Hitherto, the known number of chrysopid taxa attracted to methyl eugenol is low. Apart of *Sencera* it has been shown to attract only *Mallada basalis* (Walker) in Hawaii (Suda and Cunnington 1970) and a species of *Cunctochrysa* Hölzel in the Philippines (Umeya and Hirao 1975). During a recent field trip to Ghana in 2014 large numbers of males of an unidentified species of *Parankylopteryx* were observed similarly attracted to methyl eugenol and terpinyl acetate (IUPAC: 2-(4-Methyl-3-cyclohexen-1-yl)-2-propanyl acetate) (Martin Hauser, Stephen Gaimari pers. obs.). Other chemicals that have been found to attract male chrysopids are methyl salicylate for *Chrysopa nigricornis* (Burmeister) (James 2003) and iridol for *Chrysopa oculata* Say (Chauhan et al. 2007). This raises the question as to whether these chemicals are analogous in structure and result in similar physiological and behavioral responses for the animals.

Such observations raise many questions, including whether or not such a chemical association is pervasive across the clade comprising *Parankylopteryx*, *Ankylopteryx* *s.str.*, and *Sencera*, and whether such attraction might even represent a synapomorphy for this or a more inclusive group. It is important to investigate whether males of species of *Ankylopteryx* are attracted to this chemical and if this can be found throughout Ankylopterygini. More importantly, it remains to be discovered what the true biological significance of this trait is. Given that the baits only attract males, one immediately wonders whether these are components of semiochemicals produced by the females or if they play some other role in courtship and mating behaviors (Aldrich et al. 2009). On the surface it seems as though these may act to gather males into mating leks, from which either a chemical cue or mere location attracts females. It is possible that methyl eugenol attracts males because they in turn use it to produce female attractants, as seen in fruit flies. As of yet, this remains untested but represents a significant area of chemical ecology and behavior for investigation. Moreover, as alluded to above, discovery of the specific attractants involved for both males and females offers the possibility of amassing material for once ‘rare’ or ‘uncommon’ taxa, as well as hitherto unknown species, much as was the case when similar chemicals were found to lure male orchid bees (e.g., Dodson et al. 1969; Dressler 1982). Future collecting trips should target sampling with varied baits to see how broadly across chrysopids they are attractive and/or whether different taxa are attracted to different chemicals.

## Supplementary Material

XML Treatment for Sencera

XML Treatment for Ankylopteryx (Sencera) anomala

## References

[B1] Aldrich JR Le TC Zhang Q Torres J Winterton SL Han B Miller GL Chauhan KR (2009) Prothoracic gland semiochemicals of green lacewings. Journal of Chemical Ecology 35(10): 1181–1187. doi: 10.1007/s10886-009-9701-x 19844760 10.1007/s10886-009-9701-x

[B2] Aspöck U Aspöck H (2008) Phylogenetic relevance of the genital sclerites of Neuropterida (Insecta: Holometabola). Systematic Entomology 33(1): 97–127. doi: 10.1111/j.1365-3113.2007.00396.x

[B3] Barnard PC (1984) Adult morphology related to classification. In: Canard M Séméria Y New TR (Eds) Biology of Chrysopidae. Junk, The Hague, Netherlands, 19–29 [total volume, x+294 pp.]

[B4] Brauer F (1864) Entomologische Beiträge. Verhandlungen der Kaiserlich-Königlichen Zoologisch-Botanischen Gesellschaft in Wien 14: 891–902.

[B5] Brooks SJ (1983) A new genus of Oriental lacewings (Neuroptera: Chrysopidae). Bulletin of the British Museum of Natural History (Entomology) 47(1): 1–26.

[B6] Brooks SJ (1997) An overview of the current status of Chrysopidae (Neuroptera) systematics. Deutsche Entomologische Zeitschrift, Berlin (N.F.) 44: 267–275.

[B7] Brooks SJ Barnard PC (1990) The green lacewings of the world: a generic review (Neuroptera: Chrysopidae). Bulletin of the British Museum of Natural History (Entomology) 59(2): 117–286.

[B8] Chauhan KR Levi V Zhang Q-H Aldrich JR (2007) Female goldeneyed lacewings (Neuroptera: Chrysopidae) approach but seldom enter traps baited with the male-produced compound iridodial. Journal of Economic Entomology 100(6): 1751–1755. doi: 10.1093/jee/100.6.1751 18232390 10.1603/0022-0493(2007)100[1751:fglnca]2.0.co;2

[B9] Dodson CH Dressler RL Hills HG Adams RM Williams NH (1969) Biologically active compounds in orchid fragrances. Science 164(3885): 1243–1249. doi: 10.1126/science.164.3885.1243 17772561 10.1126/science.164.3885.1243

[B10] Dressler RL (1982) Biology of the orchid bees (Euglossini). Annual Review of Ecology and Systematics 13: 373–394. doi: 10.1146/annurev.es.13.110182.002105

[B11] Grimaldi DA Engel MS (2007) Why descriptive science still matters. BioScience 57(8): 646–647. doi: 10.1641/B570802

[B12] James DG (2003) Field evaluation of herbivore-induced plant volatiles as attractants for beneficial insects: methyl salicylate and the green lacewing, *Chrysopa nigricornis*. Journal of Chemical Ecology 29(7): 1601–1609. doi: 10.1023/A:1024270713493 12921438 10.1023/a:1024270713493

[B13] Leblanc L Rubinoff D Vargas RI (2009) Attraction of nontarget species to fruit fly (Diptera: Tephritidae) male lures and decaying fruit flies in traps in Hawaii. Environmental Entomology 38(5): 1446–1461. doi: 10.1603/022.038.0513 19825300 10.1603/022.038.0513

[B14] Nakahara W (1955) New Chrysopidae from Formosa. Kontyû 23(4): 143–147, +3 pls.

[B15] Navás L (1924 [1925]) Comunicaciones entomológicas. 7. Neurópteros del Museo de Berlín. Revista de la Real Academia de Ciencias Exactas, Físico-Químicas y Naturales de Zaragoza, 1a Serie 9: 20–34. [Date of publication 16 June 1925]

[B16] Navás L (1929) Insectos exóticos neurópteros y afines del Museo Civico de Génova. Annali del Museo Civico di Storia Naturale Giacomo Doria 53: 354–389.

[B17] Navás L (1930) Insectos del Museo de París [5.^a^ série]. Brotéria, Série Zoológica 26: 5–24.

[B18] New TR (1980) A revision of the Australian Chrysopidae (Insecta: Neuroptera). Australian Journal of Zoology, Supplementary Series 28(77): 1–143. doi: 10.1071/AJZS077

[B19] New TR (1989) Planipennia: Lacewings. In: Fischer M (Ed.) Handbuch der Zoologie: Eine Naturgeschichte der Stämme des Tierreiches, IV Band: Arthropoda: Insecta. Teilband 30 Walter de Gruyter, Berlin, Germany, 132 pp.

[B20] New TR (2003) The Neuroptera of Malesia [Fauna Malesiana Handbook 4]. Brill, Leiden, The Netherlands, viii+204 pp.

[B21] Pai KF Chen CJ Yang JT Chen CC (2004) Green lacewing *Ankylopteryx exquisite* [sic] attracted to methyl eugenol. Plant Protection Bulletin 46(1): 93–97. [In Chinese, with English abstract]

[B22] Suda DY Cunningham RT (1970) *Chrysopa basalis* captured in plastic traps containing methyl eugenol. Journal of Economic Entomology 63(5): . doi: 10.1093/jee/63.5.1706

[B23] Tan KH Nishida R (2012) Methyl eugenol: its occurrence, distribution, and role in nature, especially in relation to insect behavior and pollination. Journal of Insect Science 12(56): 1–74. doi: 10.1673/031.012.5601 10.1673/031.012.5601 PMC3500151 22963669

[B24] Tsukaguchi S (1995) Chrysopidae of Japan (Insecta, Neuroptera). Privately published, Osaka, Japan, [ii]+224 pp.

[B25] Umeya K Hirao J (1975) Attraction of the jackfruit fly, *Dacus umbrosus* F. (Diptera: Tephritidae) and lace wing, *Chrysopa* sp. (Neuroptera: Chrysopidae) by lure traps baited with methyl eugenol and cue-lure in the Philippines. Applied Entomology and Zoology 10(1): 60–62.

[B26] Winterton SL Brooks SJ (2002) Phylogeny of the apochrysine green lacewings (Neuroptera: Chrysopidae: Apochrysinae). Annals of the Entomological Society of America 95(1): 16–28. doi: 10.1603/0013-8746(2002)095[0016:POTAGL]2.0.CO;2

[B27] Winterton SL Guek HP Brooks SJ (2012) A charismatic new species of green lacewing discovered in Malaysia (Neuroptera, Chrysopidae): the confluence of citizen scientist, online image database and cybertaxonomy. ZooKeys 214: 1–11. doi: 10.3897/zookeys.214.3220 22936863 10.3897/zookeys.214.3220 PMC3426877

[B28] Yang X Yang J Li W (2005) Fauna Sinica: Insecta, Volume 39: Neuroptera: Chrysopidae. Science Press, Beijing, China, xiii+398 pp, +4 pls. [In Chinese, with English summary]

